# Effects of Topical Steroids on Postoperative Pain in Patients Undergoing Stand-Alone Lateral Lumbar Interbody Fusion

**DOI:** 10.7759/cureus.106975

**Published:** 2026-04-13

**Authors:** Soji Tani, David M Dalton, James J Hunker, Jiaqi Zhu, Jennifer Shue, Gary A Fantini, Darren R Lebl, Frank P Cammisa, Federico P Girardi, Alexander P Hughes, Gbolabo Sokunbi, Andrew A Sama, William D Zelenty

**Affiliations:** 1 Spine Surgery, Hospital for Special Surgery, New York, USA; 2 Orthopaedic Surgery, Showa University School of Medicine, Tokyo, JPN; 3 Biostatistics Core, Hospital for Special Surgery, New York, USA

**Keywords:** lateral lumbar interbody fusion, pain management, spine surgery, standalone llif, topical steroid

## Abstract

Background

Postoperative pain in the psoas and femoral nerve distribution is common following lateral lumbar interbody fusion (LLIF) surgery. Topical steroids have shown beneficial effects in spinal surgery. The effect of topical steroids applied directly to the psoas and the traversing nerve complexes in patients undergoing stand-alone LLIF (SA-LLIF) is unclear.

Purpose

The purpose of this retrospective cohort study is to investigate the efficacy of topical steroids in reducing postoperative pain when applied to the psoas muscle during SA-LLIF.

Methods

Patients who underwent SA-LLIF were included. Patient demographics, perioperative factors, length of stay (LOS), opioid consumption measured in oral morphine equivalents (OME), and pain scores on the numeric rating scale (NRS) were recorded and analyzed. Univariable and multivariable regression analyses were performed to assess the impact of topical steroid use on OME, NRS, and LOS. Subgroup analysis was conducted on patients who did not receive transversus abdominal plane (TAP) blocks.

Results

There was no significant difference between patients who received topical steroids and those who did not. However, in the subgroup analysis of those without tap block, NRS in the first 24 hours post-surgery was significantly lower in the topical steroids group (4.1 (3.0, 5.4) vs. 5.3 (3.9, 6.4) (p = 0.03)), while there was no significant difference in OME and LOS. In multivariable analysis, topical steroid use was an independent factor associated with decreased NRS in the first 24 hours post-surgery score (β = -0.74 (95% CI -1.22, -0.25)) (p < 0.01).

Conclusions

Topical steroids were associated with a lower 0-24-hour NRS score in patients who did not receive a TAP block. However, overall, topical steroids did not significantly improve pain measures during the postoperative period for patients undergoing SA-LLIF.

## Introduction

Lateral lumbar interbody fusion (LLIF) is a commonly performed surgical procedure for the treatment of various spinal disorders [1,2]. Through ligamentotaxis and disc height restoration, the LLIF approach is utilized to treat lumbar disease as an alternative to direct decompression of neurological components and posterior stabilization [1]. Unlike the conventional posterior approach, LLIF is performed via a minimally invasive retroperitoneal, trans-psoas approach. In LLIF, postoperative pain or psoas dysfunction may result from direct injury to the psoas muscle, femoral/obturator nerve, or lumbar plexus [3,4]. There may also be sensory deficits from numerous sensory nerves that pass through the psoas [3,5]. There are several reported approach- and lumbar plexus-related postoperative symptoms, including pain, paresthesia, and motor deficits [2,3,5]. Postoperative pain in the psoas and femoral nerve distribution can negatively impact patient outcomes, recovery, and quality of life [3,5,6] and increase postoperative opioid consumption [6]. It is recognized that using opioids after surgery and experiencing their negative side effects might delay a patient's recovery [7,8]. Therefore, multimodal analgesia care is being increasingly instituted in spine surgery, and nonopioid pain management strategies are of significant research interest [6,9].

Representing one component in pain management, corticosteroids reduce pain and soft tissue swelling by inhibiting the production of inflammatory prostaglandins and cytokines [10]. In orthopedic surgery, steroids are commonly used after major joint surgery and have been found to be an effective form of perioperative pain management [11,12]. Topical steroid administration has demonstrated benefits in spine surgery [13-15]. In lumbar spine surgery, epidural steroids can significantly improve postoperative pain, reduce length of stay (LOS), and decrease opioid consumption [16,17]. Topical steroids after anterior cervical surgery can decrease dysphagia and postoperative pain scores in the early postoperative phase [10,14].

Despite the proven efficacy in other instances, the potentially beneficial anti-inflammatory effect of directly applying steroids to the psoas and nerve complexes in LLIF has not been systematically investigated. We hypothesized that topical steroid application to the psoas muscle would confer improvements in postoperative pain, LOS, and opioid consumption. Therefore, this study aims to investigate the association between topical steroids and postoperative pain, LOS, and opioid consumption in patients undergoing stand-alone LLIF (SA-LLIF).

## Materials and methods

Patient population

This is a single-center, retrospective observational study. This was approved by the hospital's institutional review board (IRB #2020-1877) and conducted in accordance with the principles of the Helsinki Declaration. Consecutive patients who underwent SA-LLIF between November 2016 and July 2020 were retrospectively reviewed. The exclusion criteria were combined anterior and posterior fusion, only L5/S1 level fusion, history of prior lumbar fusion, surgery for infection or tumor, and lumbar corpectomy.

Surgical procedure and topical steroid

The surgical procedure was conducted by a previously described method [2,6]. Briefly, patients were positioned in the lateral decubitus (LLIF) and the DaVinci supine position (anterior lumbar interbody fusion). Our facility uses a mini-open or muscle-preserving approach to access the intervertebral disc. After the skin incision, the abdominal wall musculature is bluntly separated in line with the fibers. The retroperitoneal space is then developed with direct palpation. The psoas muscle is identified and divided bluntly to access the disc space. After fluoroscopic confirmation of the appropriate level, a minimally invasive retractor is docked and dilated at the segment. A standard discectomy, endplate preparation, trialing, and implant insertion through the dilating retractor is performed under fluoroscopic control. After wound irrigation, some patients received topical steroids (methylprednisolone, 40 mg) and local anesthetic (bupivacaine, 10 mg) applied directly to the psoas muscle belly. The decision to perform topical steroids was determined by and at the discretion of the treating surgeon. Some patients received a transversus abdominal plane (TAP) block based on consensus between the anesthesiologist, surgeon, and patient. For TAP blocks, 0.5% bupivacaine (20-30 mL) with 2 mg dexamethasone was injected into the abdominal layers [6]. All surgery was performed by one of six board-certified spine surgeons.

Data collection

Data were collected from the electronic medical record. Demographic variables included age, gender, body mass index (BMI), race, and smoking status. Medical comorbidities were classified using the American Society of Anesthesiologists Physical Status (ASA) classification, the Charlson Comorbidity Index (CCI) score, and a history of psychiatric illness (anxiety, depression). Medical diagnosis of chronic pain and preoperative opioid use were also recorded. Surgery-related factors included diagnosis, revision surgery, approach side, operative level, number of levels fused, surgery duration minutes, and estimated blood loss (EBL). The presence of a TAP block was also reviewed, as well as the presence of intravenous steroids administered.

The study outcomes were opioid consumption (converted to oral morphine equivalents, OME in mg/day) (intraoperative, 24 hours post-surgery, and 24-48 hours post-surgery), numeric rating scale (NRS) pain score (post-anesthesia care unit (PACU), from PACU discharge to 24 hours post-surgery, and 24-48 hours post-surgery), and LOS.

Statistical analysis

Statistical analysis was performed between the topical steroid group and the non-topical steroid group. Continuous variables were expressed as median with interquartile range (IQR). Categorical variables were expressed as numbers with percentages (%). Continuous variables were analyzed by the Mann-Whitney U test. Categorical variables were analyzed by the χ^2^ test or Fisher's exact test. A subgroup analysis was carried out without TAP block patients, according to a previous LLIF and TAP block study [6].

A series of linear regression analyses was conducted for NRS, opioid consumption, and LOS. Covariates in the multivariable regression model - topical steroid, age, inclusion of L4/5, surgery time, and CCI - were defined from clinically significant factors and factors isolated by previous studies [3,5,6]. All statistical analyses were performed with JMP Pro version 16 software (SAS Institute Inc., Cary, NC, USA). All statistical tests were two-tailed, and a p-value < 0.05 was considered statistically significant.

## Results

A total of 115 patients were eligible for this study: 56 (48.7%) patients received topical steroids, and 59 (51.3%) patients did not receive topical steroids. Baseline characteristics and surgical details of both patient groups are listed in Table 1. The study population included 69 (60.0%) males with a median age of 60.0 (52.0, 69.0) years and a BMI of 27.5 (24.4, 31.9) kg/m^2^. A total of 42 (36.5%) patients had previous lumbar decompression surgery, and 81 (70.4%) patients received the right-side approach. The number of patients who underwent SA-LLIF stratified by the number of levels fused was as follows: single level, n = 55 (47.8%); two levels, n = 43 (37.4%); three or more levels, n = 17 (14.8%). Nearly all patients (97%) received IV steroids (4-10mg of IV dexamethasone) as part of their anesthetic regimen; of those who did not, 50% received topical steroids; all four patients were included in their respective cohorts. Significant differences existed between the two groups; the topical steroid group was older, more likely to have a left-sided approach, longer surgery time, and fewer received a TAP block. Demographic differences were controlled using multivariable regression. There were no significant differences in univariate analyses of each outcome (Table 2).

**Table 1:** Baseline patient characteristics (including TAP block) Bold values indicate significance (p < 0.05). DF: degrees of freedom; BMI: body mass index; ASA: American Society of Anesthesiologists; CCI: Charlson Comorbidity Index; DM: diabetes mellitus; EBL: estimated blood loss; TAP: transversus abdominal plane; IQR: interquartile range

Variables	Category	With topical steroid	Without topical steroid	P-value	DF	Cramer’s V
Number of patients (n)	115	56	59	-	-	-
Age (years)	Median (IQR)	62.5 (55.0, 72.8)	58.0 (49.0, 68.0)	0.014	-	-
Sex, n (%)	Male	31 (55.4)	38 (64.4)	0.322	1	0.092
Race, n (%)	White Caucasian individuals	44 (78.6)	55 (93.2)	0.034	2	0.213
	Black or African individuals	4 (7.1)	1 (1.7)			
	Other	8 (14.3)	3 (5.1)			
BMI (kg/m^2^)	Median (IQR)	26.58 (24.1, 31.1)	27.80 (25.3, 32.3)	0.372	-	-
Current smoking, n (%)	-	2 (3.5)	5 (8.5)	0.439	1	0.103
ASA, n (%)	1	1 (1.8)	7 (11.9)	0.084	2	0.211
	2	44 (83.9)	47 (79.7)			
	3	8 (14.3)	5 (8.5)			
CCI, n (%)	0	6 (10.7)	15 (25.4)	0.066	3	0.250
	1	14 (25.0)	13 (22.0)			
	2	8 (14.3)	13 (22.0)			
	3 or more	28 (50.0)	18 (30.5)			
Comorbidity, n (%)	Anxiety or depression	12 (21.1)	15 (25.4)	0.577	1	0.047
	DM	4 (7.1)	4 (6.8)	1.000	1	0.007
Perioperative opioid use, n (%)	-	10 (17.5)	11 (18.6)	0.878	1	0.010
Prior operation, n (%)	-	22 (38.6)	20 (33.9)	0.599	1	0.056
Approach laterality, n (%)	Right side	30 (53.6)	51 (86.4)	<0.001	1	0.360
Operating level, n (%)	T12/L1	0 (0)	1 (1.7)	1.000	-	-
	L1/2	0 (0)	5 (8.5)	0.057	-	-
	L2/3	11 (19.6)	14 (23.7)	0.595	-	-
	L3/4	38 (67.9)	29 (49.2)	0.042	-	-
	L4/5	45 (80.4)	52 (88.1)	0.251	-	-
Number of operating levels, n (%)	1	23 (41.1)	32 (54.2)	0.016	-	-
	2	28 (50.0)	15 (25.4)			
	3	5 (8.9)	12 (20.3)			
Surgery time (min)	Median (IQR)	79 (62, 119)	55 (37, 105)	0.004	-	-
EBL (mL)	Median (IQR)	50.0 (50.0, 100)	50.0 (25.0, 100.0)	0.309	-	-
TAP block, n (%)	-	20 (35.7)	33 (56.3)	0.023	-	-

**Table 2:** Comparison of outcomes between the topical steroid and non-topical steroid groups (including TAP block) OME: oral morphine equivalents; NRS: numeric rating scale; IQR: interquartile range; TAP: transversus abdominal plane; PACU: post-anesthesia care unit

Variables	With topical steroid	Without topical steroid	P-value
Length of stay (hours) (median (IQR))	55.0 (32.7, 78.5)	54.4 (33.4, 77.2)	0.628
Intraoperative OME (median (IQR))	1.4 (0.3, 13.1)	5.0 (0.9, 12.5)	0.167
0-24 hours post-surgery OME (median (IQR))	44.8 (23.8, 97.5)	75.8 (30.0, 101.2)	0.292
24-hours post-surgery OME (median (IQR))	56.3 (9.3, 157.1)	54.4 (6.9, 166.7)	0.960
NRS at PACU (median (IQR))	5.9 (4.0, 7.3)	5.9 (4.5, 7.0)	0.829
NRS 0-24 hours post-surgery (median (IQR))	4.3 (3.2, 5.6)	4.1 (3.7, 6.0)	0.219
NRS 24- hours post-surgery (median (IQR))	4.4 (2.7, 6.0)	3.9 (2.8, 5.4)	0.566

In a post-hoc subgroup analysis of 62 patients who did not receive a TAP block, univariable analysis showed that patients who received topical steroids were older and more likely to undergo a right-sided approach (Table 3). Application of topical steroids was also associated with significantly lower NRS score within 24 hours post-surgery in this cohort (Table 4). Multivariable analysis showed topical steroids were an independent factor in a decreased 24-hour post-surgery NRS score (β = -0.74 (95% CI -1.22, -0.25)) (Table 5).

**Table 3:** Baseline patient characteristics (TAP block excluded) Bold values indicate significance (p < 0.05). DF: degrees of freedom; BMI: body mass index; ASA: American Society of Anesthesiologists; CCI: Charlson Comorbidity Index; DM: diabetes mellitus; EBL: estimated blood loss; TAP: transversus abdominal plane; IQR: interquartile range

Variables	Category	With topical steroid	Without topical steroid	P-value	DF	Cramer's V
Number of patients (n)	62	36	26	-	-	-
Age (years)	Median (IQR)	64.5 (57.3, 72.8)	58.5 (50.5, 68.0)	0.022	-	-
Sex, n (%)	Male	19 (52.8)	14 (53.8)	0.934	1	0.011
Race, n (%)	White Caucasian	32 (88.9)	24 (92.3)	0.588	2	0.094
	Black or African	3 (8.3)	1 (3.9)			
	Other	1 (2.8)	1 (3.9)			
BMI (kg/m^2^)	Median (IQR)	28.0 (23.1, 32.7)	30.4 (26.1, 35.0)	0.201	-	-
Current smoking, n (%)	-	1 (2.8)	3 (11.5)	0.300	1	0.176
ASA, n (%)	1	15 (41.7)	13 (50.0)	0.819	2	0.090
	2	17 (47.2)	10 (38.5)			
	3	4 (11.1)	3 (11.5)			
CCI, n (%)	0	2 (5.6)	6 (23.1)	0.145	3	0.298
	1	7 (19.4)	7 (26.9)			
	2	7 (19.4)	4 (15.4)			
	3 or more	20 (55.6)	9 (34.6)			
Comorbidity, n (%)	Anxiety or depression	8 (22.2)	9 (34.6)	0.280	1	0.137
DM	-	4 (11.1)	1 (3.9)	0.388	1	0.132
Perioperative opioid use, n (%)	-	6 (16.7)	8 (30.8)	0.190	1	0.166
Prior operation, n (%)	-	16 (44.4)	8 (30.8)	0.304	1	0.139
Approach laterality, n (%)	Right side	19 (52.8)	21 (80.8)	0.023	1	0.289
Operating level, n (%)	T12/L1	0 (0)	1 (3.9)	0.419	-	-
	L1/2	0 (0)	0 (0)	-	-	-
	L2/3	8 (22.2)	4 (15.4)	0.746	-	-
	L3/4	23 (63.9)	13 (50.0)	0.274	-	-
	L4/5	30 (83.3)	24 (92.3)	0.450	-	-
Number of operating levels, n (%)	1	15 (41.7)	13 (50.0)	0.819	-	-
	2	17 (47.2)	10 (38.5)			
	3	4 (11.1)	3 (11.5)			
Surgery time (min)	Median (IQR)	86.5 (59.5, 118.00)	51.5 (36.0, 119.8)	0.052	-	-
EBL (mL)	Median (IQR)	50.0 (50.0, 100.0)	50.0 (25.0, 100.0)	0.566	-	-

**Table 4:** Comparison of outcomes between the topical steroid and non-topical steroid groups (TAP block excluded) Bold values indicate significance (p < 0.05). OME: oral morphine equivalents; NRS: numeric rating scale; IQR: interquartile range; TAP: transversus abdominal plane; PACU: post-anesthesia care unit

Variables	With topical steroid	Without topical steroid	P-value
Length of stay (hours) (median (IQR))	55.0 (35.4, 93.9)	68.9 (46.2, 85.8)	0.754
Intraoperative OME (median (IQR))	1.1 (0.3, 16.3)	1.3 (0.3, 4.4)	0.694
0-24 hours post-surgery OME (median (IQR))	38.2 (23.1, 114.4)	89.4 (31.4, 115.2)	0.127
24-hours post-surgery OME (median (IQR))	52.5 (11.0, 147.5)	161.8 (30.1, 213.9)	0.076
NRS at PACU (median (IQR))	5.3 (4.0, 7.2)	6.4 (5.1, 7.6)	0.131
NRS 0-24 hours post-surgery (median (IQR))	4.1 (3.0, 5.4)	5.3 (3.9, 6.4)	0.023
NRS 24-hours post-surgery (median (IQR))	4.4 (2.7, 6.0)	4.6 (2.7, 5.9)	0.692

**Table 5:** Multivariable analyses (TAP block excluded) Bold values indicate significance (p < 0.05). CCI: Charlson Comorbidity Index; OME: oral morphine equivalents; NRS: numeric rating scale; TAP: transversus abdominal plane; CI: confidence intervals

Factor	Length of stay (hours)	Lower 95% CI	Upper 95% CI	P-value
Topical steroid	-6.22	-18.31	5.86	0.307
Age	-0.27	-1.86	1.32	0.738
Including L4/5	0.63	-17.82	16.55	0.941
Surgery time	0.33	0.08	0.59	0.010
CCI	7.73	-7.68	23.13	0.319
Factor	0-24 hours OME	Lower 95% CI	Upper 95% CI	P-value
Topical steroid	-11.36	-33.68	10.96	0.258
Age	-0.67	-3.61	2.26	0.745
Including L4/5	-26.25	-57.99	5.48	0.167
Surgery time	0.11	-0.36	0.57	0.649
CCI	5.05	-23.40	33.50	0.719
Factor	NRS 0-24 hours	Lower 95% CI	Upper 95% CI	P-value
Topical steroid	-0.74	-1.22	-0.25	0.004
Age	-0.04	-0.11	0.02	0.180
Including L4/5	-1.08	-1.77	-0.38	0.003
Surgery time	0.01	-0.004	0.017	0.198
CCI	0.44	-0.18	1.06	0.162

## Discussion

This is the first study to investigate the efficacy of topical steroids directly, specifically applied to the psoas and nerve plexus in SA-LLIF patients. In this study, the effect of topical steroids on postoperative pain, LOS, and opioid consumption in patients undergoing SA-LLIF was investigated. While no significant differences were observed in the univariate analyses, subgroup analysis of patients who did not receive TAP blocks revealed a significant decrease in NRS pain scores within 24 hours post-surgery in the topical steroid group.

The novel finding of this study is that the use of topical steroids was associated with a statistically significant reduction in postoperative pain scores within 24 hours in patients who did not receive TAP blocks, suggesting that topical steroids may be beneficial in managing acute postoperative pain following SA-LLIF. Notably, however, the observed reduction in NRS score (β = -0.74) may not represent a clinically significant difference, highlighting an opportunity for further study.

While previous studies have demonstrated the benefits of topical steroids in reducing nerve symptoms and dysphagia in various surgical procedures [13,14], their specific effect in SA-LLIF patients has not been investigated. The rationale for using steroids post lumbar decompression or anterior cervical discectomy and fusion (ACDF) differs from that for lateral lumbar surgery. In ACDF, the objective is to reduce swelling of the esophagus and the surrounding soft tissue structures that affect swallowing [14]. In posterior lumbar surgeries, steroids presumably reduce inflammation of the target nerve and peridural fibrosis and scarring at the root level in the foramen or the canal [13,18]. In LLIF, direct damage to the psoas muscle, femoral/obturator nerve, or lumbar plexus may lead to postoperative pain or psoas dysfunction [3-5]. Therefore, the postoperative sequelae of LLIF differ from ACDF and posterior lumbar surgery for several reasons. The nerves encountered during LLIF are mainly at the nerve level rather than root level and have different histological features, including a perineurium, and therefore may be less susceptible to inflammation and scarring following manipulation [19]. Injury at the nerve level may come from indirect retraction since retractors usually have a buffer of muscular tissue between them and the nerve, unlike a dural retractor placed directly on a nerve root over a disc. In injuries to the lumbar plexus, the nerves may be compressed against the transverse processes and result in a crush-type injury rather than traction. Injury to the plexus may occur as a traction injury during positioning rather than retraction, and may occur at a level away from the direct operative field. The fusion and pro-inflammatory effect of bone morphogenic protein that is commonly used in LLIF surgery may overpower any effect the steroid may have [20]. Furthermore, the field is more open, and it is challenging to localize the steroid in the area of interest.

The significant decrease in postoperative pain scores within 24 hours in patients who received topical steroids supports the hypothesis that topical steroids applied directly to the psoas and nerve complexes may have an anti-inflammatory effect, contributing to pain reduction. The observed pain reduction may be attributed to the anti-inflammatory actions of topical steroids in the surgical site, resulting in decreased soft tissue inflammation and subsequent pain [10,11]. This effect may have been attenuated in our cohort by the near-universal administration of IV steroids intraoperatively. The evidence regarding the efficacy of steroids in spinal surgery is mixed at best [7,14]. Although the evidence regarding ACDF and dysphagia rates is relatively strong, the results regarding steroids in the lumbar spine are inconsistent. There are recent articles examining steroid use in minimally invasive lumbar surgery that reviewed the current literature [13,17]. The authors highlighted the difficulty in using pain scores to evaluate perioperative pain, the heterogeneity of other measures, including LOS and opioid consumption, potential differences in the type of steroid used, and the lack of support for using steroids in meta-analysis. Their study focused primarily on decompression, while evidence for fusion surgery is less prevalent.

Conversely, there is some evidence that topical steroids can be neurotoxic and affect infection and spinal fusion rates, while other studies show no effect [21]. Our cohort had no perioperative side effects resulting from the topical steroid application.

The potential benefit of steroids should be balanced against their adverse effects. The adverse effects of systemic steroids have been extensively reported and discussed in the medical literature [22]. The local adverse effects that spine surgeons would be most concerned about are (1) non-union, (2) infection, and (3) neurotoxic effects. The effect of steroids on spinal fusion is debated and has been previously studied, showing a detrimental effect on fusion rates. Most clinical studies, particularly in the cervical spine, show no long-term adverse effects [14,23]. There is evidence of increased infection risk if fusion is performed within 30 or 90 days of an epidural steroid injection [23]. However, studies involving topical steroids at the time of surgery in the lumbar or cervical spine have not shown an increased risk of infection [13,14]. There were no identified adverse effects in our cohort. The steroid used in our study is a particulate matter steroid. Although no difference was shown between particulate and non-particulate steroids used in epidural injections, particulate steroids have potential advantages [24]. A particulate injection is thought to have a prolonged duration of action (up to 21 days) due to reduced solubility. The Food and Drug Administration (FDA) warned against using particulate matter steroids for epidural injections due to the perceived risk of postoperative paralysis [25]. The cause is disputed, and various possible pathomechanisms have been proposed, including vasospasm, emboli, and neurotoxicity [24,25]. In the current study, the steroid injection is done outside the canal and not directly on the thecal sac. Any benefit shown in the study seems to be in the first 24 hours, so the advantage of using particulate steroids is likely minimal.

One of the principal confounders in this series is whether the patients had a TAP block. Post-hoc subgroup analysis revealed a reduction in early pain scores among those who did not receive a TAP block. Previously published literature from our group showed improvement in opioid consumption and LOS in a similar and overlapping cohort in those who received a TAP block [6]. The published technique involved injecting both local anesthetic and steroids into the abdominal wall musculature. The presence of a steroid in this block means those who received a block may have had, in effect, a locally applied steroid. After the tissue planes are disrupted during the surgical approach, the steroid is likely to seep down onto the psoas. We may have a more accurate sense of topical steroids' effect after removing those who had a TAP block from the analysis. When excluding patients who had a TAP block, patients who received topical steroids had a significantly lower mean NRS score in the first 24 hours. Although this post-hoc subgroup analysis may not have been the primary outcome measure of the study, it provides insight into the potential benefit of topical steroids in lateral procedures. It also gives some insight into the potential effect size, which will be helpful in potential future studies. We should also consider the effects of systemically administered steroids, which are not accounted for in our study. Previous literature on the effect of steroids on outcome after discectomy has shown that there is a benefit to steroid administration, and the size of that effect does not change whether the steroids were administered topically or systemically [17]. Steroids are frequently given as a part of the perioperative patient anesthetic regimen to help manage postoperative nausea and vomiting. Notably, 97% of our patients received IV steroids as a component of their anesthetic regimen. Of the four patients who did not receive IV steroids, two received topical steroids. Therefore, the administration of IV steroids may have attenuated the effect of topical steroids in our study.

There are several limitations to acknowledge. Firstly, the retrospective nature of the study design introduces inherent biases and limits the ability to establish causality. The decision to administer topical steroids was based on the surgeon's discretion, leading to potential selection bias. A particularly notable potential confounder is the concomitant administration of bupivacaine with topical steroids, which may have provided some early pain relief. Furthermore, there may be confounders that were not accounted for, and variations in surgical technique among different surgeons may have affected the outcomes. The study population included patients with varying baseline characteristics and involvement of different lumbar vertebral levels, which adds to the heterogeneity of the cohort. Second, the sample size may not have been sufficient to demonstrate clinically significant differences. A larger sample size would be required for more definitive evidence. Another limitation is the lack of long-term follow-up, which restricts the assessment of sustained pain relief and other long-term outcomes of interest, such as non-union. The short-term nature of the study's observation period limits the understanding of the prolonged effects of topical steroids. Although this study provides valuable insights into the impact of topical steroids on postoperative pain in patients undergoing SA-LLIF, further prospective studies with standardized protocols and larger sample sizes are needed.

## Conclusions

In conclusion, our observational study supports that there may be some benefit in applying steroids locally to those undergoing lateral lumbar surgery, particularly in early pain reduction. However, there may not be an additional benefit if a TAP block is performed. The disadvantages of using steroids are debatable, and topical application is unlikely to have a large systemic effect.
